# Railway Line Occupancy Control Based on Distance Determination Sound Method

**DOI:** 10.3390/s22135003

**Published:** 2022-07-02

**Authors:** Rafał Burdzik, Ireneusz Celiński, Maciej Kłaczyński

**Affiliations:** 1 Faculty of Transport and Aviation Engineering, Silesian University of Technology, 40-019 Katowice, Poland; ireneusz.celinski@polsl.pl; 2 Department of Mechanics and Vibroacoustics, Faculty of Mechanical Engineering and Robotics, AGH University of Science and Technology, Mickiewicza 30, 30-059 Krakow, Poland; maciej.klaczynski@agh.edu.pl

**Keywords:** sound based distance determination, sound level, detection, rail vehicle

## Abstract

The purpose of this research paper is to present the application of the developed sound method as a supporting tool to deal with railway traffic flow control. It is found that controlling railway line occupancy is the main issue associated with railway traffic flow. For this purpose, the line occupancy control based on a sound method has been developed. The concept of using sound waves as a source of information about approaching people, animals, vehicles, etc., has been known for centuries, and is due to the natural properties of the sense of hearing. There are many engineering attempts on the use of this phenomenon, which are mostly based on applications of distributed fiber-optic sensing technology. This paper presents the results of the sound pressure measurement in the immediate proximity of the rail to analyze and evaluate the use of the acoustic wave as an information carrier on approaching rail vehicles. The purpose of this research is to discuss the sound method introduced here, and apply it in different circumstances.

## 1. Introduction

Transport systems are characterized by a disproportionate increase in demand in relation to the supply of the transport network. Increasing the demand capacity by a larger number of means of transport with constant infrastructure and the size of the transport network is not a suitable solution, because it generates congestion and delays, which slow down the total traffic flow. This is especially important in road transport, but also in rail transport. It is very important that deviations from the schedule be detected and corrected as early as possible, the regulation is used to stabilize traffic and thereby minimize passengers’ waiting times at stations and help to synchronize vehicle arrivals at connecting nodes, thus minimizing transfer delays [1]. Therefore, more and more research is being carried out in the field of vehicle flow detection, counting, and control systems in the transport network. New sensor and data transfer technologies are very helpful in this regard. The paper [2] explores the advancement of a smart traffic management system using the Internet of Things (IoT). The IoT provides an effective way of tracing interactions among web devices with the traffic embedded sensors, services, actuators, and other interconnected networks. For urban traffic, a mixture of static and dynamical systems for measuring traffic flow, such as lidar, camera, and sensor are employed. These data are continuously recorded and sent to a control management system for processing. In order to increase the reliability of the vehicle detection system, it is advisable to use various sources of information based on various physical phenomena (induction, image, vibration, sound). Big Data algorithms are applied to further enhance the intelligence of applications in the transportation field. It has a wide range of applications including signal recognition, object detection, traffic flow prediction, travel time planning, and travel route planning [3]. An extensive knowledge overview on big data algorithms and applications in the Intelligent Transportation System (ITS) is presented in [3]. Vehicle manufacturers are developing in-vehicle sensors that support safety, traffic management, and emissions. Institutions for the management of national roads and highways are implementing roadside infrastructures, such as cameras and sensors, to collect data on environmental and traffic conditions. The authors of the paper [4] discuss how sensor technology can be integrated with transportation infrastructure to achieve sustainable ITS. Not just the road transport system, but also the movement of trains on the railway network, due to the need to conduct them in a smooth, safe, and stable manner, must be properly structured. This arrangement is defined as the organization of railway traffic [5]. As one of the innovative methods of train detection, the vibration-based method can be considered. The paper [6] presents some experiments on the information capacity of the vibration signal generated by railway mechanisms. An interesting approach to train detection using vibration signal has been presented by the authors in [7]. When considering the application of a vibration signal, the acoustics phenomena should be also considered. Thus, the paper [8] presents research on identification of the vibration-acoustic signature of a moving train for the possible application in a train detection system.

The novel application of acoustics methods e.g., for aircraft noise during acoustic monitoring [9,10] or diagnosis of selected laryngeal diseases [11] were studied. The literature on railway noise are mostly focused on the environmental and human impact, especially for cargo trains and high speed (TGV). However, an important scope of the research on railway noise is the quantification of aeroacoustics and rolling noise sources emitted by trains externally. For the rolling noise, the phenomenon corresponds with the tribological properties of rail steel or wheel-rail contact forces, especially during braking, which also have to be considered [12,13,14]. It is corresponded with material vibration propagation properties [15]. For the complex analysis of phenomena influenced by rolling noise, even rail fastening should be considered, the paper [16] presents a study on numerical analysis of spring rail fastenings. The paper [17] presents a comprise source localization using two-dimensional acoustic array measurements and an assessment of the wayside noise increase as a function of the speed. The paper [18] describes the aerodynamic noise radiation from a vestibule side door on a high-speed train surface calculated by the combination of unsteady incompressible fluid flow analysis and acoustic analysis. Other groups of research are focused on the noise impact. Sleep disturbance by rail vehicle and road noises was studied through in situ physiological recordings in two groups of people submitted to both types of exposure in the article [19]. The train noise in a station is identified by a series of train noises occurring while a train arrives, stops, and leaves a station. The article [20] presents a study on determining the acoustical characteristics of train noise in stations, according to architectural elements such as location and different platforms styles. Although some studies compared the train noise propagated by various types of trains to largely free sound propagation [21,22]. The above-described attempts are an example of a negative approach to the propagation of a sound wave on and around the railway route. As every phenomenon, it has its positive aspects described in this article. The sound wave can be not only harmful, but it can also be a source of useful information for example for safety and other purposes.

Mechanisms associated with the interaction of wheel and the rail dominate the noise production of railway operations at conventional speeds and remain significant (but not dominant) even for high-speed trains. This wheel/rail noise may be divided into three main categories. Rolling noise occurs on a straight track and is predominantly caused by undulations of the wheel and rail surfaces that induce a vertical relative vibration. Impact noise can be considered as an extreme form of rolling noise that occurs at discontinuities in the wheel or rail surface. Squeal noise, occurring on sharp radius curves, is usually induced by a lateral excitation mechanism [23]. In the context of the article, it should be remembered that the detection of the approaching rail vehicle is limited on the sharp radius curves. Linear detection methods such as microwave radars and optical measurements are not adequate for this case. In this context, the presented method becomes extremely important.

An interesting direction of investigations on the application of noise and vibration methods is driving vehicle detection and classification. The paper [24] presents vehicle detection and classification systems using distributed fiber-optic acoustic sensing technology. On the basis of phenomena, distributed acoustic signals from an arbitrary point can be detected and located through this technology which can provide fully distributed acoustic information along the entire fiber link [25]. Vehicle detection methods to identify the change in the measured vibration value of the sensor corresponding to the vehicle and calculate driving time have been presented in [26]. The method presented in [24] applied a double threshold algorithm using the combination of short-term energy and short-term zero-crossing rate for detection. This algorithm has been modified in order to segment a voice signal in study of Kumar et al. [27]. Another modification of this algorithm for the purpose of vehicle detection and classification has been presented by Liu et al. in [24]. The paper [28] presents another application of distributed acoustic sensing technology for the purpose of identifying moving traffic sources based on ground vibrations. The energy of ambient noise was concentrated between 5 and 25 Hz to calculate noise cross-correlation functions, which falls into the typical traffic noise frequency band. When considering rail-vehicle detection directly, the research presented in [29] shows concept on settings real-time train tracking from distributed acoustic sensing data. Authors present an algorithm that extracts the positions of moving trains for a given point in time from vibration signals. The method presented contains the Principal Component Analysis (PCA) and pre-trained Support Vector Machine (SVM) to classify the feature vector. Therefore, as can be recognized, most of the articles and research are about applications of distributed fiber optic sensing technology.

An interesting concept named seismic traffic footprint for tracking trains, aircrafts, and cars has been presented in [30]. The authors studied traffic-generated seismic noise measured by an array of 5200 geophones. The results show that spatiotemporal filtering enhances signatures of moving traffic sources. In most of the papers, the source of information about driving vehicle cause from ground vibration. However, when considering the properties of wave propagation in a solid structure, the rail as the transmitter seems to be an interesting approach. A solid, which is a rail and a substructure and railway embankment, can be considered an elastic center. A moving rail vehicle causes the external force to be directed to the track which causes stresses in the structure and consequent deformation. The stresses generated in the rail and substructure creates an opposite force to the force generated by the passing rail vehicle. This force returns the elastic structure (rail) to the original geometric parameters. The effect of force generation on the elastic structure is the formation of a wave as a disturbance spreading within certain limits [6,31,32,33,34]. The acoustic wave is generated by the rails, whose parameters depend on such factors as: mass of moving rail vehicle(s), its velocity, technical condition (especially suspension), structure of the composition of the train, substructure condition, air temperature, soil temperature, humidity, and other (huge number) parameters. This wave disappears with increasing distance from the track in which it is generated by the moving rail vehicle. This wave also interferes with the waves coming from other rail vehicles. An important advantage of useful application of noise and vibration generated by moving vehicles is changing the approach to understanding acoustic and vibration signals only as burdensome and negatively affecting the environment. Of course it has to be underlined that railway traffic causes problems with noise and ground-borne vibrations, especially in areas where the ground is composed of clay [35]. When considering ground-borne noise and vibration from railway vehicles, exposure to human annoyance is often considered [36,37]. All these issues are extremely important, especially in the case of monitoring traffic noise in a large urban area [38].

When considering railway line occupancy control systems, the reliability is fundamental factor [39,40]. One of the solutions to increase the reliability of systems is the use of independent sources of information and decision algorithms. This paper presents the results of the sound pressure measurement in the immediate proximity of the rail to analyze and evaluate the use of the acoustic wave as an information carrier on approaching rail vehicles. The purpose of this research was to developed a sound-based distance determination method. 

## 2. Materials and Methods

**Figure 1:**
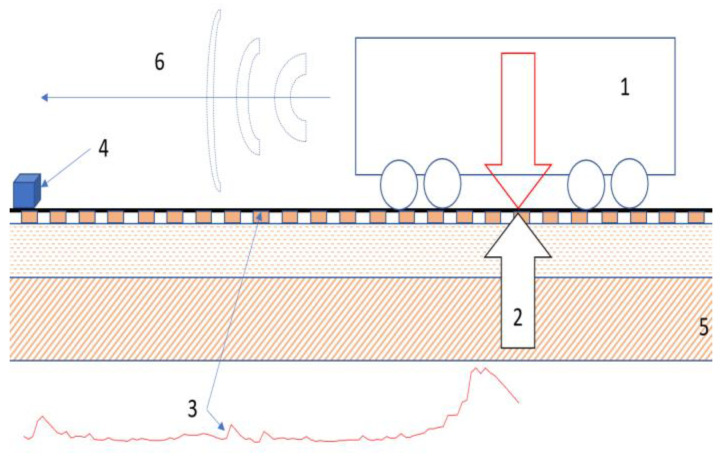
The basic idea of the experiment (1—rail vehicle, 2—force reaction, 3—rolling noise, 4—sound level meter, 5—soil and rail substructure, 6—aeroacoustics noise).

Due to the research goal as an analysis on the possibility of using an acoustic wave as an information carrier on an approaching rail vehicle, the research methodology was developed. Figure 1 presents the idea of the experiment and the concept of noise measurement and determination of the location of rail vehicles in railway network. Aeroacoustics (6) and rolling noises (3) are generated by the driving train (1). 

The idea of the proposed methodology is to measure the acoustic waveform at a certain distance from the moving rail vehicle. The purpose of the measurement is to test the possibility of identifying the position (or/and velocity) of the rail vehicle in the railway network and to assess the functionality of the presented methodology. The greatest distance from the location of the sound meter to rail vehicle position is desired: “the bigger, the better”.

Two elementary sound level meters were used: Voltcraft SL-451 and Benetech GM 1356 equipped with electret microphones. For preliminary research, such devices were used due to requirements of the EN 61 672-1 for the measurement in terrain (outside) conditions. The main assumption of the presented investigation was to analyze differences in sound level distribution collected by these two devices and based on conclusions set the regulations for final method. Thus at this stage of the investigation precise accuracy of time and phase synchronization was not the issue, but of course based on the conclusions it will be required for the final method. The calibration of the microphones was carried out for two calibration signals with the same frequency, differing between the levels 94 and 114 dB, respectively. 

The sound pressure level (SPL) was measured at selected points of the railway network. The dBA sound pressure level was measured using a filter with a correction curve A, which corresponds to the frequency bands characteristics of human audibility. 

The purpose of measuring the position of rail vehicle is to use this knowledge about the distance to specific railway infrastructure. For example, this distance is used to calculate the warning time at the level crossing.

The passage of the EN 57 passenger train running on a regular passenger line was examined. Two sound level meters were placed at distance 12 [m] from each other and 0.6 [m] from the rail head and 1.4 [m] from central axis of the track and 0.2 [m] above the head of rail level (1.435 [m] standard). 

The distance between the sound level meters was set due to the length of the rail used at the measurement place. The first sound level meter was mounted between two rail elements, transferring vibrations from the approaching rail vehicle. In this place, the biggest deformation of the course is created during the passing of the rail vehicle (with the correctly made substructure). The second sound level meter was mounted halfway along the length of the rail. The purpose of such a configuration of the measurement system was also to study the effect of the placement of measurement device on the obtained measurement results. Applications in this area require more extensive testing due to the different lengths of rails used on the network and various techniques for joining rails and performing a subgrade.

**Figure 2:**
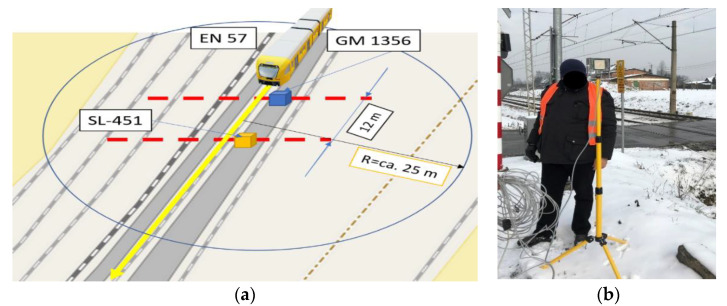
Measurement system (**a**) setup of measurement system (background maps from OSM) (**b**) measurement of rail vehicle speed using microwave radar MFDR.

The first sound level meter GM 1356 was placed next to rails connection and the second SL-451 was placed in the half the length of the rail. The location of the measurement system and its configuration is shown in Figure 2a.

For the measurement of the rail vehicle speed, the microwave radar sensor MFDR (Multi Frequency Doppler Radar) was used (Figure 2b). It uses the Doppler effect to detect the motion of objects. The microwave transmitter sends a signal, the receiver receives its reflected echo, and the detector systems process the received signal, so that after processing, they get information about the speed and direction of movement, as well as the magnitude of the object’s displacement.

**Figure 3:**
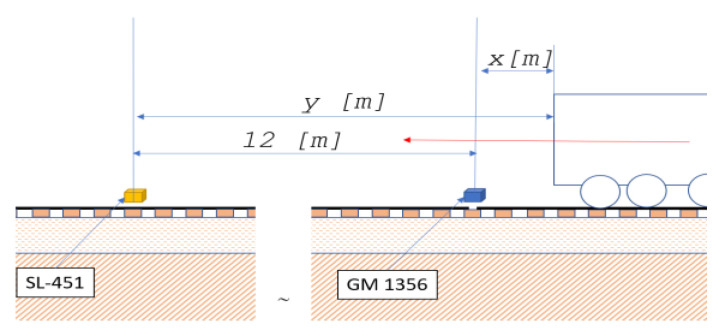
Position of sound level meters due to train driving direction.

During the research, the sound pressure levels were recorded each with a 125 m/s logger step. The average values were calculated over an interval of 1 second. The passage of the EN 57 passenger compartment operating on a regular passenger line was examined (filled rail vehicle). The distance between device GM 1356 and SL 451 is calculated in the direction of travel of the EN 57 rail vehicle. During the test in a radius of approx. 25 m from the middle of the distance between sound level meters, no devices and objects generating different noise were recorded (early morning, calm hours) (Figure 3).

## 3. Results

As far as the constant distance between sound level meters is 12 m and the rail vehicle at the same time is in the distances X from GM 1356 and Y from SL-451 (it was assumed that both signals are quasi synchronous), and it is moving at constant velocity, both sound level meters will show a real time sound level as follow:

L*_p_*_1_ = f(X(t),V(t),M) [dB] (1)

and

L*_p_*_2_ = f(Y(t),V(t),M) [dB]

where:

X—Distance from GM1356

Y—Distance from SL-451

V—rail vehicle velocity [km/h]

M—mass of the rail vehicle (constant at the measuring section) [t]

p1— refers to GM 1356

p2— refers to SL-451

**Figure 4:**
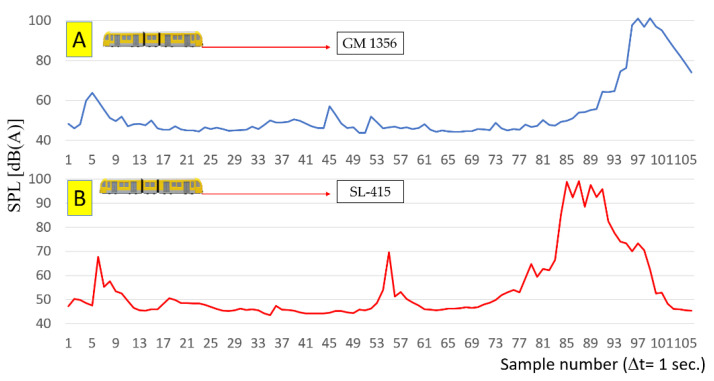
Distribution of sound level, (**A**) sound level meter GM 1356 (*p*_1_—point), (**B**) SL-415 (*p*_2_—point 2).

Equation (1) does not include factors such as substructure stiffness, air temperature and subsoil, and many others. The mass of train EN 57 is about 125 t, the speed of travel over the microphone is in range 10 km/h to 20 km/h on braking phase. The distribution of sound level measured on both sound level meters is depicted in Figure 4.

It can be observed in Figure 4 that the sound level is increasing due to the rail vehicle approaching the sound meters. The magnitude of the increase is around 50 [dB] on the measuring section 1100 [m]. It can be approximated by an exponential curve over the whole variability range; however significant deformations are observed in its course. At the same time, some local increasing section, with local maximum values, are observed. This is due to the non-uniformity of the rail and substructure.

**Figure 5:**
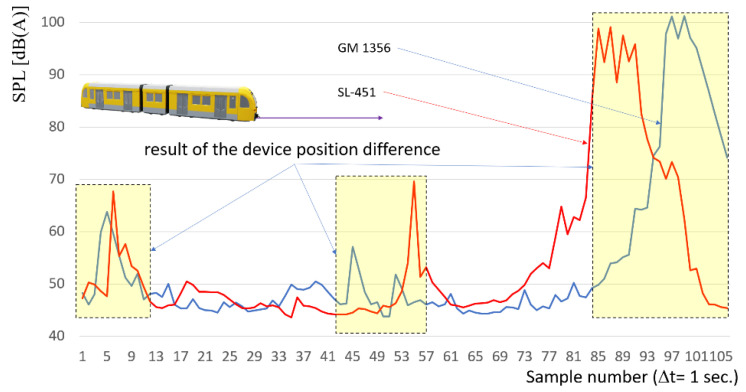
Comparison of the sound level distribution without time-shift correction. Red color line—SL-451, Blue color line—GM 1356.

The comparison of the sound level distribution without time or shift correction as a result of distance between these two sound level meters is presented in Figure 5.

Thus, the first step of the analysis was recalculated due to time versus distance differences using the equation:

(2) $$t_{s}=d_{sm}/v_{t}\;\left[s\right]$$

where:

*t_s_*—time shift,

*d_sm_*—distance between sound level meters,

*v_t_*—constant speed of the rail vehicle.

**Figure 6:**
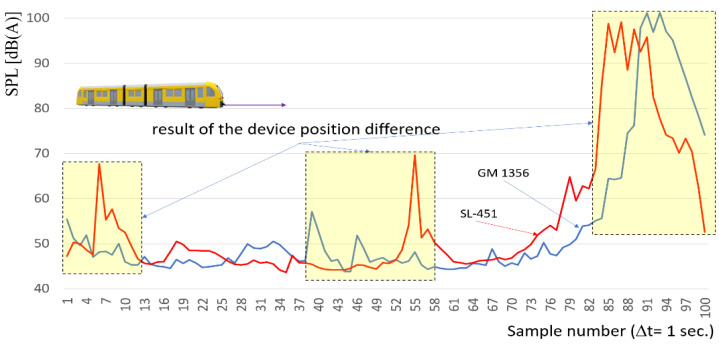
Comparison of the sound level distribution with the time shift correction. Red color line—SL-451, Blue color line—GM 1356.

As the result the acoustic pressure function with the correction due to different distance from first and second sound level meters and rail vehicle have been depicted in Figure 6. The time shift was 5 s.

Based on a comparison of the sound level distribution with the calculated (Equation (2)) time shift correction, it can be noted that the measurement system should be synchronized, not only quasi-synchronous, so it should be performed by a data acquisition device. Also, results show that more factors influence noise level than that was assumed by Equation (1). But the quality of results is sufficient for purpose of preliminary distance determination of approaching rail vehicles. The acoustic wave generated by the rail vehicle changes dynamically depending on the position of the rail vehicle. The authors currently use a GPS system for this purpose mounted at the head of rail vehicle (NMEA protocol). In addition to data from the GPS system, to identify the distance of the rail vehicle face from the first measuring point, authors currently use a prototype of a microwave radar sensor (Figure 2b). The use of such a train positioning system (microwave radar and GPS) enables the calibration of the obtained results. Such control of the obtained data is possible in the range from 50 to 700 m for medium range radar and much larger range with less accuracy for GPS. Susceptible elements, especially in rail connection area, generate local changes in the signal. The sound level meter, placed in a location susceptible to deformation of GM 1356, shows significant differences in the values of the measured characteristics in relation to a sound level meter located halfway along the length of the rail. Thus, probably a better recommendation is to locate the sound level meter at half the length of the rail. Hence, it can be hypothesized that both of the analyzed microphones locations have different functionalities for detecting the position of a rail vehicle or ruptures of the rail.

**Figure 7:**
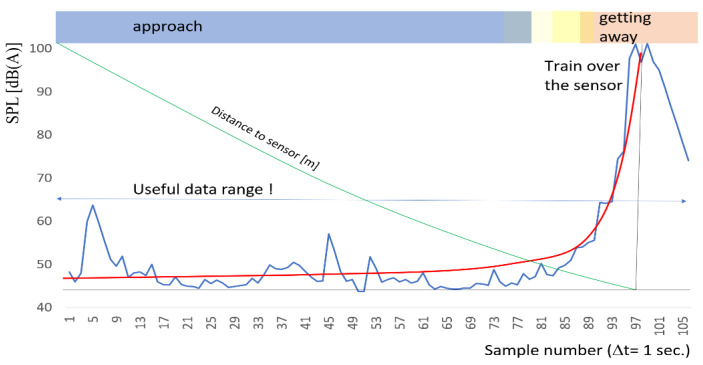
Characteristic of sound pressure level as a function of distance from the sensor (microphone).

The rail vehicle approaching the microphone area, from the moment the signal is detected until the sensor passes, generates a sound pressure level similar to the exponential distribution (red line in Figure 7, from the first sample to the sample of maximum value). At the same time, the train vehicle is approaching and the distance to the sensor decreases (green line in Figure 7). Of course the decrease distance function depends on velocity (train speed).

For better quality and accuracy of the functions, the results were resampled to increase the sampling frequency. 

## 4. Results and Discussion

The purpose of this research paper is to present an application of engineering methods as supporting tools to deal with the limitations of real-object mechanical engineering research. The first observation on the basis of the obtained results is the fact that not taking into account the susceptibility of the substrate as a springy medium requires the arrangement of a series of sensors at a certain measuring distance at different places of the subgrade, for which the sound pressure levels should be averaged at the same measurement intervals. The configuration of the position of the sound level meters requires further in-depth studies depending on the purpose of the research. Therefore, measuring the level of sound pressure in the sound level meters network should measure the following value:

(3) $$L_{p^{*}}=10lg\left(\frac{1}{n}\sum_{i=1}^{n}10^{0.1\cdot L_{pi}}\right)\;[\mathrm{dB}]$$

where:

*n*—number of microphones (sound level meters) used in research [pcs]

*L_pi_*—sound pressure level for measurement network point (single microphone)

Such an approach can allow one to not use a synchronous system due to the averaging method. It can be used for different purposes due to information carried by multiple sensors, for example, for the rail track condition monitoring. Regardless of the averaging of measurement data from individual sound level meters, the sound level curve can be smoothed out. In the example shown in Figure 4 and Figure 7, a significant increase in the level of sound pressure levels takes place during ca. 7 s, until the place where the sound level meter is located at the speed of the rail vehicle about 10 km/h. This means that the increase in the sound level value allows for detection of trains at distances depending on their speed (also mass) presented in Table 1. The linear characteristic was established for the calculation, which is a considerable simplification omitting differences in the mass and length of the train vehicle. Also, the length of the train vehicle probably has a large impact on the possibility of locating the train, especially when it comes to freight traffic. Specific lengths of individual wagons (tight assembly points of trucks) seem to have a significant impact on the rail noise and vibration. The precise detection distance of the rail vehicle is determined on the basis of its speed (measured using data from the GPS system or microwave radar). The detection distance in the proposed method is calculated assuming that the rail vehicle is moving in a uniform motion at the moment of triggering a clear signal in the measured characteristic until the first measuring point passes. In the calculations presented in Table 1, a uniform motion of the rail vehicle was assumed in 7 s which will pass to the measuring point. The theoretical detection distances for different rail vehicle speed are given.

The application of the presented method can be used for the safety information system at the level crossing. The key is the distance in which this information is obtained. The bigger the better, and it is calculated as a function of the speed of a moving rail vehicle. In typical situations, this distance should protect other users of the transport system, e.g., by information about the approaching rail vehicle at a distance greater than its braking distance. For the developed preliminary method the calculated distance of 311.11 m in Table 1, means that the warning time at level crossing is 7 s for the 160 km/h velocity. 

**Figure 8:**
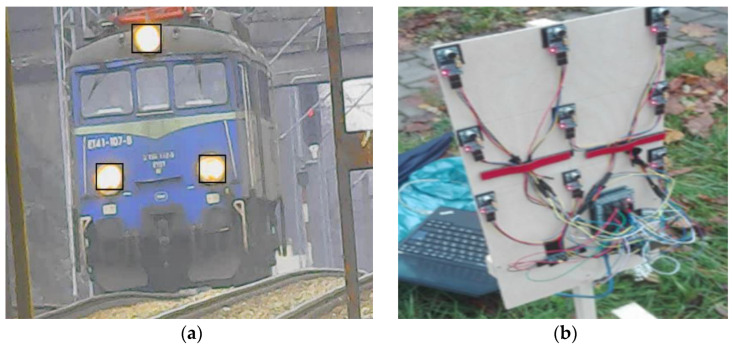
Additional (auxiliary) measurement unit: (**a**) long distance railway vehicle detection via camera (ca. 1 km from sound level meters). (**b**) short range spatial area microphone array.

Based on the analysis of the values in Table 1, it can be concluded that the study of the increase in sound pressure level, with high reliability depending on the speed of the rail vehicle. For the presented case study it is possible to monitor the railway route at a distance starting of about 20 m (for V = 10 km/h) to approx. 320 m (for V = 160 km/h). The data take into account recorded sound levels in the range of 10 to 20 km/h and do not have to increase linearly as it was included in the calculations presented in Table 1. These are therefore values that provide some information on the location of the rail vehicle on the route, but do not have practical application in the control of the current position of a rail vehicle in the context of vehicle traffic safety at the interface of the railway route - road network. This is due to the fact that the distances calculated can detect the rail vehicle are not good enough. However, if the detection distance is correlated with the train speed, it gives about 7 [s] approaching time (even for the lowest speed of 10 km/h), which is good for the preliminary investigation considered as information to the driver on level crossing. This value of approaching time is acceptable in terms of stopping or time of emergency exit from level crossing. Of course, for the real application this time has to be increased significantly. These distances can be used to calculate the warning time at most on a single track line with low maximum acceptable velocity (<80 km/h). Authors, regardless of this technique, conduct research in microwave technology and using vision techniques using special digital camera with huge optical zoom, with theoretical range up to 5 km (Figure 8a). The figure shows the detection of characteristic objects of a rail vehicle from a long distance (approximately 1000 m).

Detection of vehicle location at a large distance from the position of sound level meters enables the calibration of the rail vehicle presence measurement system in such a system. The authors managed to obtain good correlations to measure the position of rail vehicles with the use of a telephoto lens camera supported by software using vision techniques up to 2.5 km. Attempts are underway to increase the detection distance in the range from 3 to 4 km (on straight sections). Auxiliary measurement systems are also used to calibrate the system.

Authors have constructed a microphone array that allows this type of measurement with applying appropriate filters (Figure 8b). This device will be used for the further research due to final measurement method. Measuring the presence of a rail vehicle using such an acoustic matrix is different compared to many point measurements using sound level meters. In such a measurement, the sound characteristics are obtained, and visualized in the form of 3d diagrams. There is a pre-measurement; this measurement adds a value to the spot measurement. It will be described in subsequent authors’ publications. There, the additional possibility of using matrix measurements will be explained especially in the presence of many rail vehicles.

Moreover, the authors intend to install special chambers (of their own construction) in the embankment under the track in the future in order to eliminate various disturbance factors. It is also considered to use surface microphones used, for example, in automotive or laryngophonics measurements.

The devices presented in Figure 8a,b support the authors’ basic research with the use of sound levels meter.

For better results and longer distance of detection, the position of the microphone can be changed and the distance from rail can be much shorter than 0.6 m. In addition, the sound level meters can be isolated from the sounds from the external environment, which should improve its directional characteristics. Also for the further research it can be examined to narrow the width of the auditory band. This requires testing various rail vehicles in the level of range of the sound they generate. 

**Figure 9:**
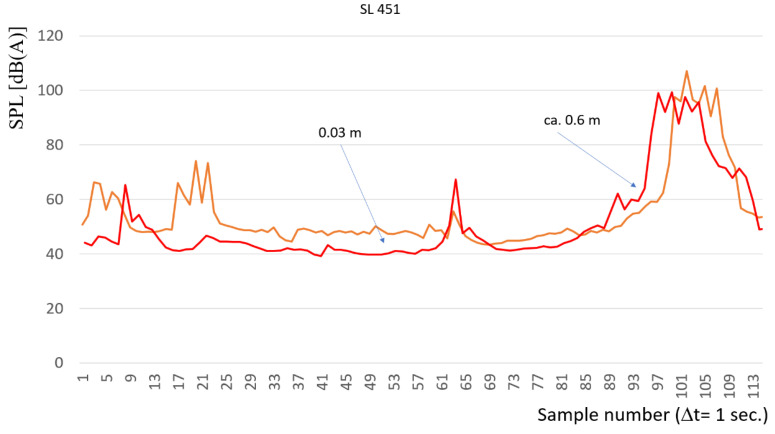
Comparison of the characteristic of the sound pressure level for different distances between the rail microphone (0.03 and 0.6 m). Red color line—distance 0.6 m, Orange color line—distance 0.03 m.

Due to first conclusion, additional research and experiments have been conducted. The purpose of an additional investigation was to examine the influence of distance between the train and microphone on the possibilities of train detection based on the acoustic signal. This study was carried out by reducing the distance of the microphone from the rail head 20 times from approx. 0.6 m to 0.03 m. The course of the sound level in this case is shown in Figure 9. Based on the analysis of Figure 9, it can be concluded that the course of the characteristics is similar in both cases for different distances of the sound level meters from the rail head—some shifts are observed depending on the distance of the rail vehicle. 

Based on the analysis of Figure 9, it can be concluded that the influence of the distance between the head of the sound level meters and the head of the rail has some significance for the values of the measurement results. This applies in particular to longer distances from the face of the train from the sound level meter microphone than several dozen meters (the first signal is received from the rail). This is very important from the point of view of the purpose of the research being carried out - it is desirable to detect the train as far as possible. 

Average values of sound pressure levels analysis of the data in both cases showed that the average level of sound pressure levels for the measurement distance of 0.03 m was 54.8 dB. For the measuring distance ca. 0.6 m, this value amounted to 54.2 dB, respectively. Therefore, reducing the 20-times distance of the sound level meter head from the measured element allowed for an average increase in the level of sound pressure by less than 2% in dB scale. Whereas in some recorded samples the difference in recorded sound level exceeded 20 dB, i.e., around 40% in dB scale. This is the basis for further in-depth analysis of rail vehicle recognition using the methodology in question. The standard deviation in both cases is almost identical (approximately 14 dB), which allows estimating the distances of the rail vehicle, regardless of the distance from the sound level meter and the head of the rail only based on the observation of the mean values of the measurement.

**Figure 10:**
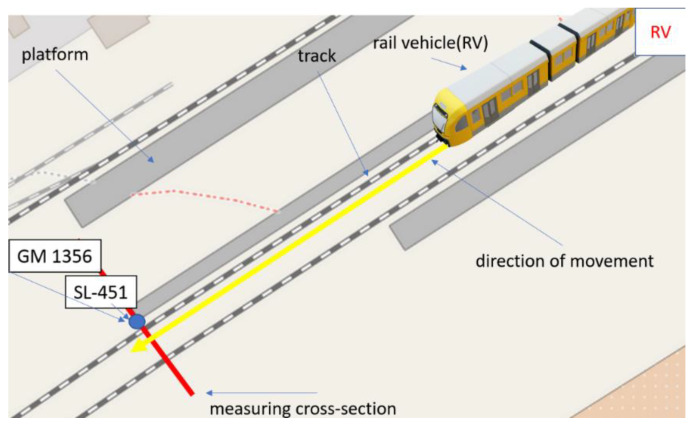
Localization of the measurement system during additional research. (Background maps from OSM).

As a result of preliminary research discussion, further additional investigations have been conducted. The scope of the research included measurements of sound level for different types of train vehicles with microphone placement on distance 0.03 m and 0.15 m from the head of rail. Two sound level meters were located at the head level behind the stopping point of the train (preliminary research focused on the location of the sound level meter in the head before the stop point of the rail vehicle). In relation to the first (preliminary) measurement, this is a significant difference, changing the course of the observed characteristics. The locations of the sound level meters are shown on the background of the OSM (Open Street Maps) map in Figure 10.

**Figure 11:**
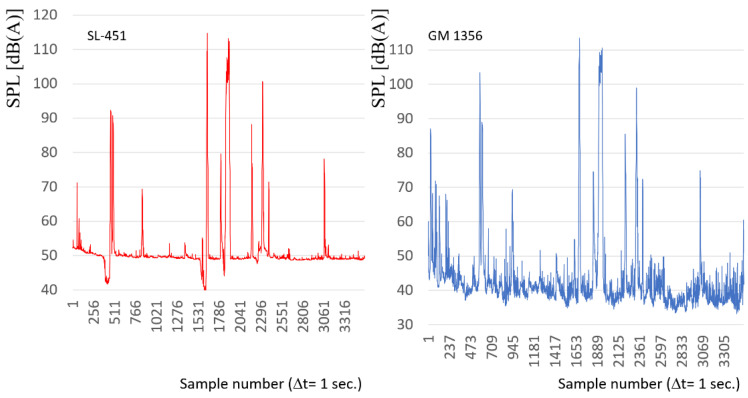
One hour characteristics of sound level distribution for both sound level meters ((**left**) SL-451, (**right**) GM-1356).

During additional research, no single runs of the rail vehicle were measured, but a continuous record was recorded in the period close to one clock hour. Unlike previous research, trains run frequently on this route, as well as trains often run in the same direction one after the other at subsequent track distances (interstate line). With this process is the connected interference of acoustic waves from the front of passing rail vehicle and interference of vibrations caused by the both vehicles in the wheel-rail system. On the route, moreover, different types of passenger trains significantly differing in their characteristics (e.g., EN75 vs. EN57) were registered. A larger number in the train designation means more modern rolling stock. The route also recorded the course of the high-speed train as well as the freight train. Total signals as one hour characteristics of sound level distribution for both sound level meters are shown in Figure 11.

During the hourly measurement of the level of sound pressure levels at the track line, the following routes of railway vehicles were registered, both on the measured track on which the sound level meters were installed and in the opposite direction (Table 2).

**Figure 12:**
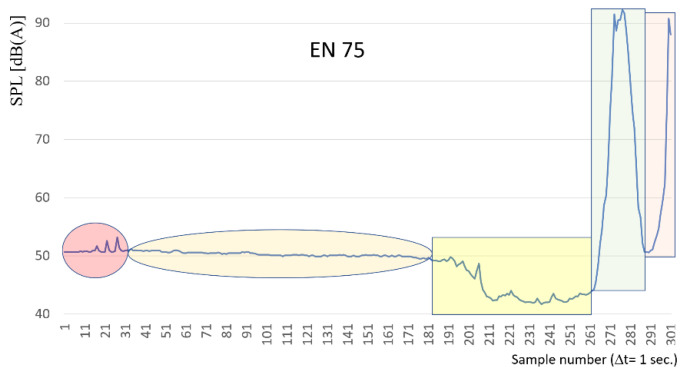
Characteristics of sound level distribution for EN 75/1 train.

As an example, the period of EN75/1 train passing has been isolated and depicted in Figure 12. The level of sound pressure first increases when the rail vehicle approaches the sound level meter, then decreases when the vehicle stops at the station, and then increases again for the vehicle moving from the place of detention. The characteristics of uniformly delayed movement (green rectangle) and uniformly accelerated motion (orange rectangle) are recorded. The rectangle in yellow indicates the area where the level of measured intensity decreases—it is probably caused by the interference of the acoustic wave coming from the train EU07 coming from the opposite direction. The characteristics presented in Figure 12 show high peaks already over 4.5 min after the train stopped at the station. For a speed of 40 km/h, this means that there is probably a place on the track at a distance of about 2.5 km from the station where the rail vehicle strongly interacts with the route (from the map it can be a railway viaduct). At a distance of 2.5 km to 1 km from the place of detention there are small peaks, which require the development of a diagnostic method with very high resolution. The interference of waves of passing trains marked in the yellow rectangle has a course dependent on the speed of movement of both railway vehicles and their physical characteristics (weight, suspension, length, etc.).

**Figure 13:**
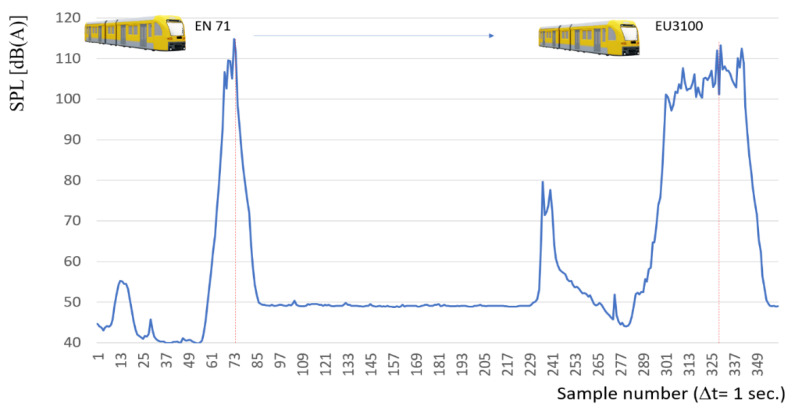
Characteristics of sound level distribution for EN71 and EU3100 trains.

Figure 13 presents an example of the results register for passing two trains (one by one, one queue). The first is the EN71 passenger train and the second EU3100 freight train. The differences in sound level distribution for the maximum range are significant.

**Figure 14:**
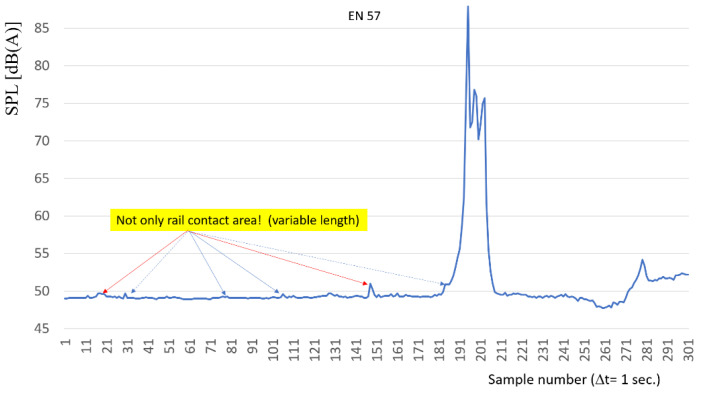
Characteristic of sound level distribution for EN57 train.

Figure 14 presents the measured characteristics for the EN 57 passenger train. In this figure, the red ovals are marked with the peak locations in the sound-level characteristics, at the earliest at a distance of about 2 km from the station. If these peaks are related to the interaction of the rail vehicle with the track and/or the substructure, the identification of their location in time and space is synonymous with the possibility of identifying the rail vehicle and, to some extent, its position in the railway line.

Due to the results of pilot studies, which were presented in this article, it can be concluded that testing the position of a rail vehicle using sound level meters in the acoustic band, however technically possible, which has been verified, does not allow the use of this method in the issues of traffic safety at level crossings. Identifiable with the use of increasing the sound level of the distance between rail vehicles and the level crossing, they do not allow the use of this information in practice to increase traffic safety. However, an additional information function may be introduced: time to head of a rail vehicle. The research presented will be developed in the direction of measuring amplitude changes in the level of sound pressure level and their frequency. In this case, it will be important to indicate the distance of rail vehicles from the location of the sound level meters, in which the change in the level of sound pressure in relation to the average level can be unambiguously deal with the appearance of the rail vehicle. In this approach, the level of changes in the average level of sound pressure is tested:

Δ*L_p_* = avg(*L_p_*(Δ*t*)) − *L_t_*(*t*) [dB] (4)

where:

$L_{p}\left(t\right)$—average sound pressure level of time interval [dB]

$L_{t}\left(t\right)$—sound level of background noise near the sound level meter [dB]

**Figure 15:**
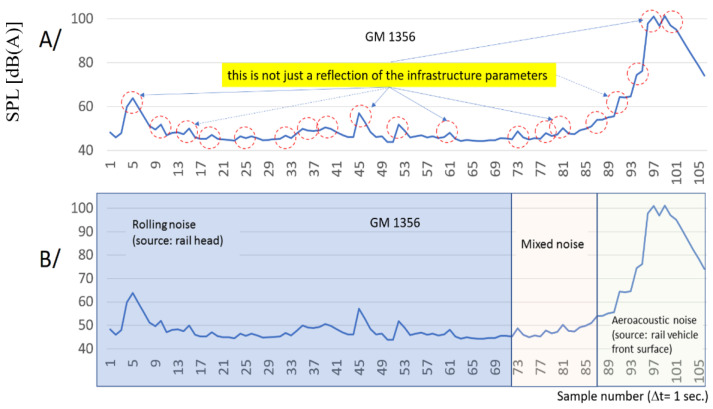
The characteristics of changes in the amplitude of the sound pressure: (**A**) as a function of the distance from the sensor, (**B**) compartments of dominant sources of rail vehicle noise.

The issue of discussion is the determination of such levels Δ*L_p_*, which uniquely identify the appearance of a train on the railway route at a set distance from the sound level meters. Based on some example results, it can be seen from the course of the characteristic that changes in signal amplitude in the conducted study are visible at distances over 1100 m from the location of the sound level meters (Figure 15A). It is difficult to verify on the basis of these pilot studies how signal frequency analysis can influence the measuring system’s ability to identify the position of a rail vehicle.

The characteristics shown in the paper are a combination of the sounds generated by the rail head and the rail vehicle itself (aeroacoustics and rolling noise). At a distance of more than one kilometer, the head of the sounds of the rail dominate. As proximity to the sensors location approaches, the signal sounds coming directly from the moving rail vehicle (aeroacoustics noise) become dominant. Figure 15B illustrates this phenomenon.

As the recommendation, after preliminary experiments, it can be given that the location of the sound level meter should be placed at half the length of the rail. This location will increase the probability of causing similar track and rail conditions to reduce any unwanted noises coming from ballasts and rail joint types and conditions. 

## 5. Conclusions and Final Remarks

Based on the measured characteristics of the sound pressure level several working hypotheses can be presented:

- The sound pressure level measurement does not have to be carried out directly at the head of the rail.
- Studies carried out indicate that the sound pressure level characteristics associated with train traffic probably concern the area of the entire embankment in the section in which the sound level meter is installed, while the location of the sound level meter heads changes the observed characteristics slightly.
- The sound propagation is associated with the appearance of the train front concerns relatively short distances of several dozen meters (maximum 100 ÷ 150) and 160 m for a freight train registered in the study.
- Peaks observed in the characteristics of sound pressure levels concern the place of occurrence on the route of engineering elements with different physical parameters than the remaining route on this section (culvert, viaduct, passage—the matter of the stiffness of the ground).
- Some peaks observed in the sound level characteristics may come from the contact of the rails that have loosened or other assembly disadvantages.
- Further in-depth tests require signal interference from trains moving from opposite directions of travel.
- A similar remark applies to trains traveling one after the other after the same section.
- Some infrastructure points are not suitable for measurement.

Due to the observed limited visibility at level crossings in Poland, even small detection times are useful in practice for selected infrastructure objects. It is worth mentioning that in recent years, there has been a tendency to reduce the frequency of trimming urban green areas (Poland). This is especially important for small objects of this type. The secondary effect is the reduction in visibility at level crossings. For low speeds on category D level crossings and regulations related to speed limits, even times from 3 s may help to increase traffic safety on these objects (at low speed). Stopping road vehicles at low speeds is possible due to new information and warning systems installed from 2021 on level crossings in Poland (manufacturer DR-Tech LLC). The system of this manufacturer increases the organizational functionality of this type of infrastructure objects via VMS signs.

Studies have shown that the noise level characteristics associated with trains are likely to affect the area of the entire embankment in the cross section in which the sound level meter is installed, while the location of the microphone heads slightly changes the observed characteristics.

The results also confirm that the emitted noise is increased with higher speeds too, but while the rolling noise rises with a speed exponent of about 3, the sound emitted by aerodynamic sources rises with a speed exponent of about 6 [38].

The authors have set up such a different sound level meter configuration relative to the rail head due to the possible snowfall, hail, storm etc. of the railway track and the exposure to pollution of the measuring system. Moreover, it is possible to isolate the microphones from other external noise (e.g., under rail chamber).

In addition, further testing may be used to narrow the width of the audio band to be tested. This requires testing various rail vehicles within the range of their sound level. It may be possible to increase the distance in which rail vehicles can be detected using this test methodology. The authors also work on the calibration of this method using microwave measurements (radar) and in vision technology (extra-zoom digital camera with footage processing). In practice, advance warning has been achieved over a distance of 1000 m using a vision system (but this is mainly applicable on straight sections of the track). It is possible to extend this detection range to 2–3 km (daylight conditions). By coupling the additional system with vision techniques, a more reliable measurement system is obtained. 

The pilot studies show the possibility of using an acoustic wave as an information carrier on an approaching train, e.g., warning time associated with lowering the gate. Thus, it can be considered as having potential applications, for example, as support for the railway safety system or monitoring system [36,37].

Changes in rolling stock, in particular the introduction of modern bogie structures, and changes in the construction of the track necessitate constant testing using the proposed technique. The threat to the presented method is the continuous modernization of infrastructure and rolling stock. The railway route is becoming more and more effective in muting. This will shorten the effective detection distances of approaching rail vehicles in the distant future. However, according to the authors, this will not be an exponential decline.

## Figures and Tables

**Table 1:** Calculated distance of detection of rail vehicle approaching.

Speed [km/h]	Speed [m/s] *	Detection Distance [m]
10	2.78	19.44
20	5.56	38.89
30	8.33	58.33
40	11.11	77.78
50	13.89	97.22
60	16.67	116.67
70	19.44	136.11
80	22.22	155.56
90	25	175
100	27.78	194.44
110	30.56	213.89
120	33.33	233.33
130	36.11	252.78
140	38.89	272.22
150	41.67	291.67
160	44.44	311.11

*—the equivalent speed is given in [m/s].

**Table 2:** Schedule of trains passing during registration time.

[hh: mm]	Train Vehicle [-]	Direction [-]	Type [-]
13:34	EN75/1	Measured ^1^	Passenger
13:34	EU07	Opposite	Passenger
13:54	EN71	Measured	Passenger
13:55	EN76/1	Opposite	Passenger
13:57	EU3100	Measured	Freight
14:03	EP09	Opposite	High speed
14:05	EN57	Measured	Passenger
14:06	EN75/1	Opposite	Passenger
14:18	EN76/2	Opposite	Passenger

^1^ Remark: Measured (measured MFDR direction), opposite (direction), two directions of movement are measured simultaneously from one device.

## Data Availability

The data presented in this study are available on request from the corresponding author. The data are not publicly available due to company policy.

## References

[1] Dridi M., Mesghouni K., Borne P. (2005). Traffic control in transportation systems. J. Manuf. Technol. Manag..

[2] Rabby M.K.M., Islam M.M., Imon S.M. A Review of IoT Application in a Smart Traffic Management System. Proceedings of the 2019 5th International Conference on Advances in Electrical Engineering (ICAEE).

[3] Kaffash S., Nguyen A.T., Zhu J. (2021). Big data algorithms and applications in intelligent transportation system: A review and bibliometric analysis. Int. J. Prod. Econ..

[4] Guerrero-Ibáñez J., Zeadally S., Contreras-Castillo J. (2018). Sensor Technologies for Intelligent Transportation Systems. Sensors.

[5] Jacyna M., Gołębiewski P., Pyza D. (2018). Railway traffic organization model considering allocation of platform edges for passenger trains. Arch. Transport. Syst. Telemat..

[6] Burdzik R., Nowak B. (2017). Identification of the Vibration Environment of Railway Infrastructure. Procedia Eng..

[7] Celiński I., Burdzik R., Młyńczak J., Kłaczyński M. (2022). Research on the Applicability of Vibration Signals for Real-Time Train and Track Condition Monitoring. Sensors.

[8] Burdzik R., Słowiński P., Prentkovskis O., Yatskiv (Jackiva) I., Skačkauskas P., Junevičius R., Maruschak P. (2022). Application of Pass-Band Step Filtering Method for Identification the Vibration-Acoustic Signature of a Moving Train. TRANSBALTICA XII.

[9] Burdzik R., Celiński I. Preliminary research and analysis on the possibility of using an acoustic wave as an information carrier on an approaching train. Proceedings of the 14th Conference on Dynamical Systems Theory and Applications.

[10] Kłaczyński M. (2014). Identification of aircraft noise during acoustic monitoring by using 3D sound probes. Acta Phys. Pol. A.

[11] Kłaczyński M. (2015). Vibroacoustic methods in diagnosis of selected laryngeal diseases. J. Vibroeng..

[12] Bąkowski H., Piwnik J. (2016). QuantItatIve and qualitative comparison of tribological properties of railway rails with and without heat treatment. Arch. Metall. Mater..

[13] Bąkowski H., Posmyk A., Krawczyk J. (2011). Tribological properties of rail steel in straight moderately loaded sections of railway tracks. Arch. Metall. Mater..

[14] Mańka A., Sitarz M. (2016). Effects of a thermal load on the wheel/brake-block subsystem: The thermal conicity of railway wheels. Proc. Inst. Mech. Eng. Part. F.

[15] Burdzik R., Lisiecki A., Warczek J., Folęga P., Szkliniarz A., Siwiec G. (2016). Research on vibration properties of copper-titanium alloys. Arch. Metall. Mater..

[16] Sładkowski A., Bizoń K. (2017). The use of semi-automatic technique of finite elements mesh generation for solutions of some railway transport problems. Mechanics.

[17] Mellet C., Létourneaux F., Poisson F., Talotte C. (2006). High speed train noise emission: Latest investigation of the aerodynamic/rolling noise contribution. J. Sound Vib..

[18] Sassa T., Sato T., Yatsui S. (2001). Numerical analysis of aerodynamic noise radiation from a high-speed train surface. J. Sound Vib..

[19] Vernet M. (1983). Comparison between train noise and road noise annoyance during sleep. J. Sound Vib..

[20] Shimokura R., Yoshiharu S. (2011). Characteristics of train noise in above-ground and underground stations with side and island platforms. J. Sound Vib..

[21] Stuber C. (1975). Air and structure borne noise of railway. J. Sound Vib..

[22] Kurze U.J., Diehl R.J., Weibenberger W. (2000). Sound emission limits for rail vehicles. J. Sound Vib..

[23] Thompson D.J., Jones C.J.C. (2000). A review of the modelling of wheel/rail noise generation. J. Sound Vib..

[24] Liu H., Ma J., Xu T., Yan W., Ma L., Zhang X. (2020). Vehicle Detection and Classification Using Distributed Fiber Optic Acoustic Sensing. IEEE Trans. Veh. Technol..

[25] Eileen M., Lindsey N., Dou S., Ajo-Franklin J., Daley T., Freifeld B., Bjella K. Interferometry of a roadside DAS array in Fairbanks, AK. Proceedings of the 2016 SEG International Exposition and Annual Meeting.

[26] He Z., Guan W., Ma S. (2013). A traffic-condition-based route guidance strategy for a single destination road network. Transp. Res. Part C Emerg. Technol..

[27] Kumar S., Phadikar S., Majumder K. (2017). Modified segmentation algorithm based on short term energy & zero crossing rate for Maithili speech signal. Proc. Int. Conf. Access. Digit. World.

[28] Zeng X., Lancelle C., Thurber C., Fratta D., Wang H., Lord N., Chalari A., Clarke A. (2017). Properties of Noise Cross-Correlation Functions Obtained from a Distributed Acoustic Sensing Array at Garner Valley, California. Bull. Seismol. Soc. Am..

[29] Wiesmeyr C., Litzenberger M., Waser M., Papp A., Garn H., Neunteufel G., Döller H. (2020). Real-Time Train Tracking from Distributed Acoustic Sensing Data. Appl. Sci..

[30] Riahi N., Gerstoft P. (2015). The seismic traffic footprint: Tracking trains aircraft and cars seismically. Geophys. Res. Lett..

[31] Lei X., Noda N. (2002). Analyses of dynamic response of vehicle and track coupling system with random irregularity of track vertical profile. J. Sound Vib..

[32] Ling L., Jin X.-S., Xiao X., Xiong J.-Y., Zhou L., Wen Z.-F. (2014). A 3D model for coupling dynamics analysis of high-speed train/track system. J. Zhejiang Univ. Sci. A.

[33] Auersch L. (2010). Theoretical and experimental excitation force spectra for railway induced ground vibration: Vehicle-track-soil interaction, irregularities and soil measurements. Veh. Syst. Dyn..

[34] Krylov V. (1997). Spectra of low frequency ground vibrations generated by high speed trains on layered ground. J. Low Freq. Noise. Vib. Act. Control.

[35] Gidlöf-Gunnarsson A., Jerson T., Ögren M., Öhrström E. (2012). Railway noise annoyance and the importance of number of trains, ground vibration, and building situational factors. Noise Health.

[36] Nering K., Kowalska-Koczwara A., Stypuła K. (2020). Annoyance Based Vibro-Acoustic Comfort Evaluation of as Summation of Stimuli Annoyance in the Context of Human Exposure to Noise and Vibration in Buildings. Sustainability.

[37] Connolly D.P., Marecki G.P., Kouroussis G., Thalassinakis I., Woodward P.K. (2016). The growth of railway ground vibration problems—A review. Sci. Total Environ..

[38] Benocci R., Roman H.E., Zambon G. (2021). Optimized Sensors Network and Dynamical Maps for Monitoring Traffic Noise in a Large Urban Zone. Appl. Sci..

[39] Siergiejczyk M., Rosiński A. (2015). The concept of monitoring a teletransmission track of the highway emergency response system. Diagnostyka.

[40] Martens A., Wedemann J., Meunier N., Leclere A., Deutsche B.A.G., Systemtechnik D.B. (2002). High speed train noise-sound source localization at fast passing trains. Dtsch. Bahn AG Soc. Esp. De Acoust..

